# The association between self-reported lack of sleep, low vitality and impaired glucose tolerance: a Swedish cross-sectional study

**DOI:** 10.1186/1471-2458-13-700

**Published:** 2013-07-31

**Authors:** Susanne Andersson, Inger Ekman, Febe Friberg, Erik Bøg-Hansen, Ulf Lindblad

**Affiliations:** 1 Institute of Health and Care Sciences, The Sahlgrenska Academy of the University of Gothenburg, Gothenburg, Sweden; 2 School of Life Sciences, University of Skövde, P.O. Box 408, 54128, Skövde, Sweden; 3 Centre for Person-Centred Care (GPCC), University of Gothenburg, P.O. Box 4571405 30 Gothenburg, Sweden; 4 Faculty of Social Sciences, Department of Health, University of Stavanger, Stavanger, Norway; 5 Institute of Medicine, Department of Primary Health Care, The Sahlgrenska Academy of the University of Gothenburg, Gothenburg, Sweden; 6 The Department of primary health care, University of Gothenburg, Box 154, Gothenburg 405 30, Sweden

**Keywords:** Impaired glucose tolerance, Fatigue, Sleeping disorders, Health conversation, Primary health care

## Abstract

**Background:**

The increased incidence of impaired glucose tolerance (IGT), are serious public health issues, and several studies link sleeping disorders with increased risk of developing type 2 diabetes, impaired glucose tolerance and insulin resistance (IR). This study explore how self-reported lack of sleep and low vitality, are associated with IGT in a representative Swedish population.

**Methods:**

A cross-sectional survey conducted in two municipalities in South-western Sweden. Participants aged 30–75 were randomly selected from the population in strata by sex and age. Altogether, 2,816 participants were surveyed with a participation rates at 76%. Participants with normal glucose tolerance (n=2,314), and those with IGT (n=213) were retained for analyses. The participants answered a questionnaire before the oral glucose tolerance test (OGTT). Associations for questions concerning sleeping disorders, vitality and IGT were analysed using logistic regression and were expressed as odds ratios (OR) with 95% CI.

**Results:**

In men a statistically significant age-adjusted association was found between self-reported lack of sleep and IGT: OR 2.4 (95% CI: 1.1-5.4). It did not weaken after further adjustment for body mass index (BMI), smoking, education, and leisure time physical activity 2.3 (1.0-5.5, p=0.044). No such associations were found in females. Corresponding age-adjusted associations between low vitality and IGT in both men 2.8 (1.3-5.8), and women 2.0 (1.2-3.4) were successively lost with increasing adjustment.

**Conclusions:**

Insufficient sleep seems independently associated with IGT in men, while low vitality was not independently associated with IGT neither in men nor women, when multiple confounders are considered. IGT should be considered in patients presenting these symptoms, and underlying mechanisms further explored.

## Background

The increased incidence of impaired glucose metabolism (IGM) [1,2] and its association with vascular complications [3] are serious public health issues. Usually, type 2 diabetes is preceded by a period of hyperinsulinemia as a consequence of insulin resistance (IR), impaired glucose tolerance (IGT) and/or impaired fasting glucose (IFG) [4]. Individuals with IFG have predominantly an increased hepatic insulin resistance and differ from those with IGT who primarily have insulin resistance in skeletal muscle [5]. Estimates predict that 60% of people who develop diabetes have IGT or IFG about 5 years earlier [6]. Sleeping disorders have adverse consequences on multiple systems [7] and several studies link sleeping disorders with IGM and increased risk of developing type 2 diabetes, IGT and IR [8-10]. The causal effects of sleep disorders and the underlying path physiological mechanisms involved have not been fully elucidated [11] and the association between diabetes, obesity and sleep disorders may be described as a vicious circle [12]. Though several behavioral factors may influence these mechanisms, low physical activity and obesity are the main confounding factors being associated with both IGM, sleep function and experienced well-being.

However, the question remains if the experience of not getting enough sleep to feel thoroughly rested is causally related to the underlying pathophysiological mechanisms in the development of IGT. The feeling of not being fully rested may also be connected to the feeling of low vitality, and fatigue may be one way to express this [13]. Interestingly, we have previously found that people with IGT similarly describe fatigue as abnormal day time sleepiness that increases the need for rest and a feeling of not being as strong as before [13]. An International Diabetes Federation expert committee also recommends professionals consider that patients with IGM may also suffer from sleeping disorders, low vitality and fatigue, and vice versa [14]. Considering that both the American Diabetes Prevention Program [15], and the Finnish Diabetes Prevention Study on IGT [16], using diet and physical activity showed dramatic effects in the prevention of overt diabetes, while similar evidence are lacking for IFG, the focus for preventive strategies is on IGT.

Our aim was accordingly to further characterise subjects with early disturbances in the glucose metabolism being at risk for development of type 2 diabetes. To avoid bias due to secondary effects we identified individuals not yet aware of their condition to explore the association of self-reported lack of sleep and low vitality in relation to IGT in a representative Swedish population.

## Methods

### Study population

**Figure 1:**
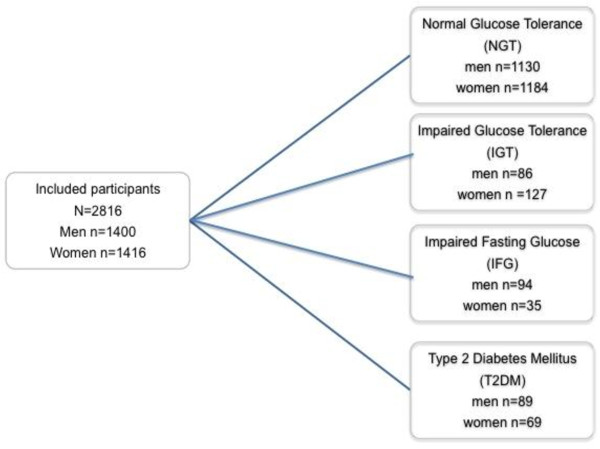
**Flowchart of the Skaraborg Project 2002–2005.** The total number of participants 2,816, and the participation rate was 76%. Two participants had incomplete OGTT, and of those with NGT or IGT 19 participants did not answer the question on lack of sleep, and 50 subjects did not respond to the question on vitality. The current study populations thus included 2508 participants (lack of sleep) and 2477 (vitality), respectively.

The present study is a cross-sectional survey carried out in 2002–2005 in two municipalities in South-western Sweden. Skaraborg County is located in the southwest of Sweden and in the year 2000 had approximately 270,000 inhabitants. The vast majority have lived in Sweden for generations and only a few per cent are immigrants from countries outside Scandinavia. Within the Skaraborg Project a survey was conducted in 2002–2005 in the municipalities of Vara and Skövde, as previously described in detail (n=2816, with a participation rate of 76%) [17]. The participants aged 30–75 were randomly selected from the population in strata by sex and age. Selected participants were invited by mail to participate in a health survey with two visits. The further selection of the current study population of participants with normal glucose tolerance (NGT) or IGT is shown in Figure 1 (n=2,314, and n=213, respectively). Two subjects had incomplete OGTT, 19 participants did not answer the question on sleep, and 50 subjects did not respond to the question on vitality. The current study populations thus included 2508 participants (lack of sleep) and 2477 (vitality), respectively (Figure 1).

### Study procedures

On the first study visit, specially educated laboratory assistants obtained blood samples in the morning after an overnight fast (10 h). Plasma glucose was analysed at the hospital laboratory at Kärnsjukhuset, Skövde, Sweden. During the OGTT, the participants were asked to answer a questionnaire covering information about medical history, current medications, socio-economic factors, smoking habits, leisure time physical activity (LTPA), self-rated health and sleeping disorders. The question regarding sleeping disorders used for this paper was: Do you think that you get enough sleep to feel thoroughly rested? The participants chose one of three alternative answers: 1. Yes, usually. 2. Yes, but not often enough. 3. No, never or almost never. The question regarding symptoms of vitality was: Have you during the last four weeks felt full of vitality? The six reply alternatives were: 1. Yes, all of the time 2. Yes, most of the time. 3. Yes, a lot of the time. 4. Some of the time. 5. No, almost never. 6. No, not at all. The question was categorized, all/most of the time, a lot of/some of the time and almost never/not at all, in 3 categories. The question regarding LTPA had four answer alternatives and low physical activity levels 1 and 2, while levels 3–4 were considered physically active [17]. Information on alcohol consumption was collected by a validated questionnaire measuring the total intake of alcohol by beer, wine, and spirits over a 30-days period [18]. A standard resting blood pressure and the anthropometric measures were measured on a second visit two weeks later by two specially trained nurses. Current smoking was defined as daily smoking (yes/no). Concentrations of serum cholesterol were determined using standard procedures. All participants were tested for fasting plasma glucose (FPG), and an OGTT was performed in all patients without a known diagnosis of diabetes. Diagnoses of IFG, IGT and diabetes were determined according to recommendations by WHO [19].

### Statistical analyses

Characteristics of participants with IGT were presented using participants with normal glucose metabolism as a reference, using SPSS Base System 19.0 for Windows for data analyses. Continuous variables were presented as age-adjusted means with SD (standard deviation). Differences in means between groups were analysed by GLM (Generalized linear model) and associations between categories were analysed using logistic regression and expressed as odds ratios (OR) with 95% CI. All comparisons between groups were adjusted for differences in age and other covariates were included when indicated. All tests were 2-sided and statistical significance was assumed at p <0.05.

### Ethical approval

The study was approved by the Regional ethical review board, Gothenburg University, Sweden (2001-10-15, Dnr. Ö 199–01) and written consent was obtained from each participant.

## Results

IGT was diagnosed in 213 participants: 86 men and 127 women (Figure 1). Table 1 shows that both men and women with IGT were older than men and women with NGT (Table 1), and they both also had a more atherogenic risk factor profile than participants with NGT. Low level of physical activity, a low level of education, and a diagnosis of hypertension were also more common in both men and women with IGT.

**Table 1:** Characteristics of 1.400 men and 1.416 women with normal glucose tolerance (NGT), impaired fasting glucose (IFG) and impaired glucose tolerance (IGT) in the Vara-Skövde cohort 2002-2005

	**NGT**		**IFG**			**IGT**		
	**(n=1130)**		**(n=94)**			**(n=86)**		
**Characteristics**	**Mean**	**(SD)**	**Mean**	**(SD)**	**p**	**Mean**	**(SD)**	**p**
*Men*								
Age (years)	45.9	(10.7)	53.4	(11.6)	<0.001	56.0	(12.3)	<0.001
SBP (mm Hg)	122	(14.3)	129	(17.2)	0.093	133	(19.4)	0.001
DBP (mm Hg)	71	(9.7)	74	(10.3)	0.157	76	(11.6)	0.021
S-Cholesterol	5.4	(1.0)	5.2	(1.0)	0.009	5.6	(1.1)	0.559
S-triglycerides	1.4	(0.8)	1.6	(0.9)	0.003	2.0	(1.6)	<0.001
S-HDL Cholesterol	1.22	(0.28)	1.14	(0.35)	0.006	1.1	(0.3)	0.004
Body mass index (kg m^-2^)	26.5	(3.3)	28.1	(3.9)	<0.001	28.3	(4.1)	<0.001
Waist hip ratio (m m^-1^)	0.93	(0.1)	0.96	(0.1)	<0.001	0.98	(0.1)	<0.001
Waist (cm)	94	(9.2)	99	(10.3)	<0.001	100	(11.1)	<0.001
	**NGT**		**IFG**			**IGT**		
	**(n=1130)**		**(n=94)**			**(n=86)**		
**Characteristics**	**n**	**(%)**	**n**	**(%)**	**p**	**n**	**(%)**	**p**
*Men*								
Lack of sleep	418	(37)	27	(29)	0.775	33	(39)	0.025
Low vitality	457	(41)	38	(40)	0.903	41	(50)	0.056
Daily smoking	168	(15)	18	(19)	0.279	11	(13)	0.638
Low physical activity	635	(58)	60	(65)	0.303	62	(77)	0.002
Hypertension	111	(9)	24	(26)	0.063	29	(34)	0.005
Living alone	222	(20)	16	(17)	0.801	30	(35)	<0.001
Low level of education	307	(28)	40	(44)	0.570	39	(49)	0.963
	**NGT**		**IFG**			**IGT**		
	**(n=1184)**		**(n=35)**			**(n=127)**		
**Characteristics**	**Mean**	**(SD)**	**Mean**	**(SD)**	**p**	**Mean**	**(SD)**	**p**
*Women*								
Age (years)	46.2	(10.9)	56.3	(11.1)	<0.001	53.7	(12.8)	<0.001
SBP (mm Hg)	117	(1.0)	131	(17.7)	0.022	133	(22.6)	<0.001
DBP (mm Hg)	68	(9.7)	72	(10.1)	0.435	74	(10.9)	<0.001
S-Cholesterol	5.1	(1.1)	5.9	(1.0)	0.066	5.5	(0.9)	0.800
S-triglycerides	1.1	(0.54)	1.7	(0.7)	<0.001	1.5	(0.7)	<0.001
S-HDL Cholesterol	1.4	(0.33)	1.3	(0.36)	0.001	1.3	(0.4)	<0.001
Body mass index (kg m^-2^)	26.1	(4.8)	30.1	(6.3)	<0.001	29.6	(6.2)	<0.001
Waist hip ratio (m m^-1^)	0.82	(0.1)	0.87	(0.1)	0.001	0.86	(0.1)	<0.001
Waist (cm)	84	(12.5)	94	(14.4)	<0.001	92	(14.5)	<0.001
	**NGT**		**IFG**			**IGT**		
	**(n=1184)**		**(n=35)**			**(n=127)**		
**Characteristics**	**n**	**(%)**	**n**	**(%)**	**p**	**n**	**(%)**	**p**
*Women*								
Lack of sleep	466	(40)	16	(47)	0.195	52	(41)	0.413
Low vitality	698	(52)	21	(60)	0.301	74	(61)	0.048
Daily smoking	246	(21)	9	(26)	0.272	23	(18)	0.706
Low physical activity	791	(69)	26	(79)	0.336	107	(86)	<0.001
Hypertension	111	(10)	7	(20)	0.872	46	(36)	<0.001
Living alone	225	(19)	6	(17)	0.381	26	(21)	0.679
Low level of education	236	(20)	18	(512)	0.228	44	(36)	0.759

*NGT* normal glucose tolerance, *IFG* impaired fasting glucose, *IGT* impaired glucose tolerance. *SBP* systolic blood pressure, *DBP* diastolic blood pressure.

Data are means and (SD), or numbers and proportions (%). IFG and IGT were compared to NGT using general linear models for continues variables, and logistic regression for categorical variables and adjusting for differences in age.

The associations between self-reported lack of sleep and IGT in men and women are shown in Table 2. The OR for IGT in men with a lack of sleep was 2.5 (1.1-5.9), when adjusted for age, BMI, smoking, low level of education, low physical activity and alcohol consumption. No corresponding association was seen in women.

**Table 2:** Association between lack of sleep and impaired glucose tolerance and impaired fasting glucose, respectively, using normal glucose tolerance as reference

	**None**	**Intermediate**		**Severe**		**None**	**Intermediate**		**Severe**	
	**OR**	**OR**	**95% CI**	**OR**	**95% CI**	**OR**	**OR**	**95% CI**	**OR**	**95% CI**
**Men**						**Women**				
**Lack of sleep and the association with IGT**										
*Adjusted for age*										
	1	1.6	(0.9-2.7)	2.4	(1.1-5.4)	1	1.2	(0.8-1.8)	1.1	(0.5-2.2)
*Adjusted for age, BMI*										
	1	1.5	(0.9-2.6)	2.3	(1.0-5.2)	1	1.1	(0.7-1.7)	1.0	(0.5-2.1)
*Adjusted for age, BMI, smoking*										
	1	1.5	(0.9-2.6)	2.4	(1.1-5.3)	1	1.1	(0.7-1.6)	1.0	(0.5-2.1)
*Adjusted for age, BMI, smoking, level of education*										
	1	1.4	(0.8-2.5)	2.6	(1.2-5.9)	1	1.1	(0.7-1.7)	1.1	(0.5-2.2)
*Adjusted for age, BMI, smoking, level of education, level of physical activity*										
	1	1.3	(0.7-2.3)	2.3	(1.0-5.5)*	1	1.1	(0.7-1.7)	1.0	(0.5-2.2)
*Adjusted for age, BMI, smoking, level of education, level of physical activity, alcohol consumption*										
	1	1.3	(0.7-2.3)	2.5	(1.1-5.9)	1	1.1	(0.7-1.7)	1.1	(0.5-2.1)
**Lack of sleep and the association with IFG**										
*Adjusted for age*										
	1	1.0	(0.6-1.7)	0.7	(0.2-1.9)	1	1.5	(0.7-3.2)	1.9	(0.6-5.9)

*IFG* impaired fasting glucose, *IGT* impaired glucose tolerance, *BMI* body mass index.

Associations were estimated using logistic regression and were expressed by OR and 95% confidence intervals.

*p=0.044.

Table 3 shows that when low and severely impaired vitality were compared to normal, significant age-adjusted associations were found between the lowest level of vitality and IGT in both men 2.8 (1.3-5.8), and women 2.0 (1.2-3.4). This association remained in men when further adjusting for BMI, smoking, or level of education but was lost when level of LTPA was entered into the model. In women, the corresponding association remained significant, but was just lost when also alcohol consumption was entered to the model.

**Table 3:** Association between low vitality and impaired glucose tolerance and impaired fasting glucose, respectively, using normal glucose tolerance as reference

	**None**	**Intermediate**		**Severe**		**None**	**Intermediate**		**Severe**	
	**OR**	**OR**	**95% CI**	**OR**	**95% CI**	**OR**	**OR**	**95% CI**	**OR**	**95% CI**
**Men**						**Women**				
**Low vitality and the association with IGT**										
*Adjusted for age*										
	1	1.4	(0.8-2.2)	2.8	(1.3-5.8)	1	1.3	(0.9-2.0)	2.0	(1.2-3.4)
*Adjusted for age, BMI*										
	1	1.4	(0.8-2.3)	2.7	(1.3-5.7)	1	1.3	(0.8-1.9)	1.7	(1.0-3.0)
*Adjusted for age, BMI, smoking*										
	1	1.4	(0.8-2.3)	2.8	(1.3-5.8)	1	1.3	(0.8-2.0)	1.8	(1.0-3.1)
*Adjusted for age, BMI, smoking, level of education*										
	1	1.3	(0.8-2.3)	2.2	(1.0-5.0)	1	1.4	(0.9-2.1)	1.9	(1.1-3.3)
*Adjusted for age, BMI, smoking, level of education, level of physical activity*										
	1	1.1	(0.6-1.9)	2.0	(0.9-4.4)	1	1.3	(0.8-2.0)	1.8	(1.0-3.2)^*^
*Adjusted for age, BMI, smoking, level of education, level of physical activity, alcohol consumption*										
	1	1.1	(0.6-2.0)	2.0	(0.9-4.6)	1	1.3	(0.8-2.1)	1.7	(0.9-3.0)
**Low vitality and the association with IFG**										
*Adjusted for age*										
	1	1.0	(0.7-1.7)	1.0	(0.4-2.3)	1	1.2	(0.5-2.5)	2.3	(0.9-5.8)

*IGT* impaired glucose tolerance, *IFG* impaired fasting glucose, *BMI* body mass index.

Associations were estimated using logistic regression and were expressed by OR and 95% confidence intervals.

*p=0.048.

We found no associations between lack of sleep and low vitality with IFG (Tables 2 and 3).

## Discussion

We found a significant association between self-reported lack of sleep and IGT in men that remained after adjustment for potential confounders such as obesity and health behaviours. No such association was found in women. Furthermore, a significant association was found between low vitality and IGT in both men and women. While the association was more robust in women, it was lost in men when other patient-related factors were considered. The study also confirmed that both men and women with IGT have a more serious cardiovascular disease risk factor profile than participants with NGT. A worse risk factor profile was also revealed in both men and women with IFG as compared to NGT, however, we found no associations with lack of sleep or low vitality.

In support of our findings, the Quebec Family Study [20] found that both short and long total sleeping time predicted type 2 diabetes/IGT in adult men and women. Clinical effects of sleep deprivation are associated with common symptoms such as sleepiness, increased fatigue, and low motivation [21], a finding that is in agreement with the results of this study. Furthermore, voluntary sleep reduction is increasingly becoming a way of life – a habit related to the modern 24 hour society [22], and studies show that nearly 30% of the middle-aged population reported sleeping less than 6.5 h per night [23]. As a consequence, reports of day-time sleepiness have become more frequent during recent years [24]. The cause of sleep-loss is multi factorial, although obesity is considered an important risk factor for obstructive sleep apnea (OSA) [25]. Accordingly, in the FIN-D2D survey [26], middle-aged men with sleep-disordered-breathing (SDB) had an increased incidence of type 2 diabetes and abnormal glucose tolerance. However, no corresponding association has been found in women in either the FIN-D2D survey [26] or the study of women in Gothenburg [27].

The significant association found between level of vitality and IGT was robust in women, but was diluted and lost in men when successively accounting for other patient-related factors. As fatigue is one of the more common presenting symptoms of type 2 diabetes, this is interesting. Physical activity came out as an explanatory factor in men, but an interaction should be expected in both genders by virtue of its strong association with insulin resistance and consequently the development of both IGT and type 2 diabetes [28]. Since physical activity is known to improve mood, and a good mood most likely increase the motivation for physical activity it may thus also be associated with the vitality factor [29].

As shown in a prospective study, psychological distress such as fatigue, anxiety and insomnia increases the risk for prediabetes and type 2 diabetes in Swedish middle-aged men [30]. There is a common belief that day-time sleepiness is normal or related to poor life style or laziness, especially if it interferes with daily functions [28]. However, OSA often characterized by daytime sleepiness, and diabetes share common mechanisms including age and obesity, but the direction of causality may go both ways [28]. Consequently, OSA should also be considered when these symptoms are investigated. The underlying mechanism also involving genetics, for example a mutation in the melatonin receptor 1B, should also be considered [31].

Our study question on sleep function did not account for sleep duration or possible SDB. However, the difference between men and women in the association between lack of sleep and IGT is consistent with the findings in the FIN-D2D study [26], and might thus be explained by the fact that men are more exposed to OSA, or more susceptible to the effects of OSA. Nevertheless, our findings were not diluted when BMI was adjusted for. Unfortunately, we did not measure breathing pattern during sleep directly or by questionnaire. In a previous Swedish study, men were also more susceptible to psychological distress in the association with prediabetes than women [30]. We found no corresponding pattern in the association with low vitality. This may probably be explained by low vitality being derived from other mechanisms than psychological distress.

### Strengths and limitations of the study

This study is based on a large, random population sample with a high participation rate, making the results generalizable to this and other similar populations. A further strength of this study is the enrolment of both men and women over a wide age-band where strategies of diabetes prevention are important. The prevalence of IGT was also congruent with other studies from Sweden [30].

An OGTT was performed in each participant without a known diagnosis of diabetes. According to recommendations from WHO, an OGTT should be performed to diagnose IGT as it is characterized by postprandial hyperglycemia and separate from IFG that is characterized by fasting hyperglycemia. This procedure to identify IGT is supported by experiences from other population studies [32]. The questionnaires were completed before the information of the results of the OGTT was available, and therefore none of the participants were aware of their potential diagnosis of IGT when answering the questionnaire. In this study we used self-reported information on physical activity on physical activity in leisure time, (LTPA). However, this question has shown good validity when recently compared to objectively measured total physical activity during 24 hour [33]. Limitations of the study comes from it’s cross-sectional design, making it impossible to decide on causality in associations. Information on sleep function and vitality was self-reported and not based on direct measurements; however, these questions have been used and validated in other studies with reliable findings. Thus, non-restorative sleep was explored in the Minimal Insomnia Symptom Scale (MISS) [34], and our question on vitality was included in a validation of the SF-36 questionnaire [35]. The question on self-experienced lack of sleep in this study is a general perception on the sleep quality covering both sleep duration and sleep quality as compared to instruments that evaluate more specific domains of sleep quality like the Pittsburg Slep Quality Index [36]. It still correlates well with other characteristics of sleep function [34]. Finally, the protocol did not comprise any measurements on OSA and HbA1c was not measured in the complete sample, and thus these important factors could not be accounted for.

## Conclusions

There is a link between self-reported lack of sleep, low vitality and IGT. Short sleep duration is a risk factor for developing type 2 diabetes [37] and voluntary sleep restriction may contribute to the global epidemic burden of type 2 diabetes [10]. More research is needed to determine how quality of sleep and low vitality interact in persons who are susceptible to developing IGT, thus facilitating improved strategies for prevention.

For health professionals, this study emphasizes the importance of paying attention to patients’ symptoms of sleep deficiency and low vitality. Patients with lack of sleep and low vitality should be considered for evaluation of a possible IGT in line with the IDF expert committee [14]. In particular, male patients with insufficient sleep and related symptoms such as low vitality should be tested generously for possible impaired glucose metabolism, using OGTT more frequently. This is correspondingly true for women expressing low vitality. Our findings are in accordance with the current strategy of opportunistic screening and primary care is the legitimate setting for primary prevention of type 2 diabetes. Listening to the patients’ symptoms may be one way of helping to identify persons at risk in order to prevent the development of type 2 diabetes.

## Competing interests

The authors declare that they have no competing interests.

## Authors’ contributions

All authors conceived of the study, and UL supervised the data collection. SA, EB-H and UL participated in the data and statistical analysis. SA has collect data, been involved in drafting the study, interpretation of data and manuscript review. UL has drafted and coordinated the study design, led the analysis of the cross sectional data and full manuscript development. IE has been involved in drafting the study, interpretation of data, manuscript review, editing for intellectual content. FF has been involved in drafting the study interpretation of data, manuscript review and editing for intellectual content. EB-H has collect data, been involved in drafting the study and the interpretation of data, manuscript review and editing for intellectual content. All authors read and approved the final manuscript.

## Pre-publication history

The pre-publication history for this paper can be accessed here:

http://www.biomedcentral.com/1471-2458/13/700/prepub
